# Geographic Accessibility and Availability of Radiotherapy in Ghana

**DOI:** 10.1001/jamanetworkopen.2022.26319

**Published:** 2022-08-11

**Authors:** Aba Anoa Scott, Alfredo Polo, Eduardo Zubizarreta, Charles Akoto-Aidoo, Clement Edusa, Ernest Osei-Bonsu, Joel Yarney, Bismark Dwobeng, Michael Milosevic, Danielle Rodin

**Affiliations:** 1 National Centre for Radiotherapy and Nuclear Medicine, Korle Bu Teaching Hospital, Accra, Ghana; 2 Radiation Medicine Program, Princess Margaret Cancer Centre, Toronto, Ontario, Canada; 3 Department of Radiation Oncology, University of Toronto, Toronto, Ontario, Canada; 4 Global Cancer Program, Princess Margaret Cancer Centre, Toronto, Ontario, Canada; 5 International Atomic Energy Agency, Vienna, Austria; 6 Oncology Department, Sweden Ghana Medical Centre, Accra, Ghana; 7 National Radiotherapy and Nuclear Medicine Centre, Komfo Anokye Teaching Hospital, Kumasi, Ghana

## Abstract

**Importance:**

Radiotherapy is critical for comprehensive cancer care, but there are large gaps in access. Within Ghana, data on radiotherapy availability and on the relationship between distance and access are unknown.

**Objectives:**

To estimate the gaps in radiotherapy machine availability in Ghana and to describe the association between distance and access to care.

**Design, Setting, and Participants:**

This is a cross-sectional, population-based study of radiotherapy delivery in Ghana in 2020 and model-based analysis of radiotherapy demand and the radiotherapy utilization rate (RUR) using the Global Task Force on Radiotherapy for Cancer Control investment framework.

**Exposures:**

Receipt of radiotherapy and the number of radiotherapy courses delivered.

**Main Outcomes and Measures:**

Geocoded location of patients receiving external beam radiotherapy (EBRT); median Euclidean distance from the district centroids to the nearest radiotherapy centers; proportion of population living within geographic buffer zones of 100, 150, and 200 km; additional capacity required for optimal utilization; and geographic accessibility after strategic location of a radiotherapy facility in an underserviced region.

**Results:**

A total of 2883 patients underwent EBRT courses in 2020, with an actual RUR of 11%. Based on an optimal RUR of 48%, 11 524 patients had an indication for radiotherapy, indicating that only 23% of patients received treatment. An investment of 23 additional EBRT machines would be required to meet demand. The median Euclidean distance from the district centroids to the nearest radiotherapy facility was 110.6 km (range, 0.62-513.2 km). The proportion of the total population living within a radius of 100, 150 and 200 km of a radiotherapy facility was 47%, 61% and 70%, respectively. A new radiotherapy facility in the northern regional capital would reduce the median of Euclidean distance by 10% to 99.4 km (range, 0.62-267.7 km) and increase proportion of the total population living within a radius of 100, 150 and 200 km to 53%, 69% and 84%, respectively. The greatest benefit was seen in regions in the northern half of Ghana.

**Conclusions and Relevance:**

In this cross-sectional study of geographic accessibility and availability of radiotherapy, Ghana had major national deficits of radiotherapy capacity, with significant geographic disparities among regions. Well-planned infrastructure scale-up that accounts for the population distribution could improve radiotherapy accessibility.

## Key Points

**Question:**

What is the treatment capacity and geographic accessibility of radiotherapy in Ghana?

**Findings:**

In this cross-sectional study of patients in Ghana eligible for external beam radiotherapy in 2020, only 23% of patients received treatment, with the highest rates of radiotherapy utilization in regions with the shortest distance to a radiotherapy facility. The median Euclidean distance from the district centroids to the nearest radiotherapy facility was 110.6 km, and only 47% of the population lived within a 100-km radial distance.

**Meaning:**

These findings suggest that there is an enormous need to scale up the availability of radiotherapy in Ghana, with consideration for the population distribution and disparities in treatment accessibility across the country.

## Teaser

This cross-sectional study estimates the gaps in radiotherapy machine availability in Ghana and describes the association between distance and access to care.

## Introduction

Radiotherapy is a critical component of comprehensive cancer treatment, but there are enormous global disparities in access.^1^ Access to radiotherapy has typically been measured as the availability of an adequate supply of machines to meet the population needs. However, access can also be affected by the distribution of health care resources.^2^ Accessibility, defined as the distance of the population to health care services within countries, is another important dimension of radiotherapy access that has been less well characterized.^3^

Geographic accessibility to health care services has been identified in many countries as an important factor affecting their utilization. Studies in breast, rectal, and prostate cancer have shown that long distances to radiotherapy facilities affect the choice of treatment given to patients,^4,5,6,7^ decrease overall radiotherapy utilization,^8^ and are associated with higher cancer mortality.^9^ These geographic disparities to radiotherapy are not limited to low- and middle-income countries (LMICs) and have also been observed in high-income countries (HICs).^8,10^

Previous estimates of radiotherapy availability, also termed radiotherapy coverage, have quantified the proportion of cancer cases needing radiotherapy based on the cancer burden and evidence-based guidelines.^1^ Recent estimates of radiotherapy availability in Africa report that current capacity is only able to meet 30% of the estimated need.^11^ Ghana, an LMIC in Africa, has demonstrated strong leadership and high-level support to improve the availability of radiotherapy.^12^ However, the shortfall in radiotherapy resources in Ghana and the accessibility of these resources to the Ghanaian population across the country are unknown.

The objective of this study was to quantify the geographic availability and accessibility of radiotherapy in Ghana at a national and regional level. We extended prior estimates of radiotherapy availability at the level of World Bank income groups or world regions to characterize utilization at the country and regional levels for both external beam radiotherapy (EBRT) and brachytherapy and the additional resources required to meet the needs of the population. We also used geospatial modeling techniques and demographic data from radiotherapy facilities in Ghana to determine the geographic accessibility of radiotherapy in different regions of the country. Finally, the outcomes of strategically located radiotherapy scale-up was evaluated.

## Methods

This cross-sectional study was approved by the institutional review boards of the Korle Bu and Komfo Anokye Teaching Hospitals and the Sweden Ghana Medical Centre. Informed consent was waived by the institutional review boards of the 3 institutions because of the retrospective nature of the study. This report follows the Strengthening the Reporting of Observational Studies in Epidemiology (STROBE) reporting guideline for observational studies.

### Setting

Ghana is an LMIC in West Africa with a gross domestic product per capita in 2020 of US $2324.^13^ It has a population of 31.1 million, with approximately 43% of the population residing in rural areas as of 2021.^14,15^ Administratively, it is divided into 16 regions, which are further subdivided into 260 districts.^15^ Three-quarters of the population lives in the 8 regions in southern Ghana, with the remainder residing in the 8 regions in the north.^15^ In the south, the most populous region is Ashanti, with 19% of the population, followed by Greater Accra, with 16%.^15^

In 2020, there were an estimated 24 009 incident cases of cancer in Ghana, and this is projected to double by 2040, largely because of aging and population growth.^16^ Breast, prostate, and cervical cancers are most frequently diagnosed and are all disease sites for which radiotherapy plays an important role in curative and palliative management.^16^ There are 3 radiotherapy facilities in Ghana, with 1 facility located in Kumasi, the Ashanti regional capital, and the other 2 in Accra, the Greater Accra regional capital, which are all used to full capacity.^12^ The facility in Kumasi has 2 EBRT machines and 1 brachytherapy afterloader. The 2 facilities in Accra have a total of 3 EBRT machines and 2 brachytherapy afterloaders. The remaining 14 regions have no radiotherapy facilities. At a national level, this translates to 0.17 megavoltage (MV) treatment units per million population, or 0.2 MV units per 1000 cancer cases.

### Data Sources

Population density data in 2020 in Ghana were obtained with 100-m spatial resolution from the WorldPop database.^17^ These data were verified with population data by region from the Ghana Statistical Service website.^15^ Cancer incidence data in 2020 in Ghana were obtained from the International Agency for Research on Cancer GLOBOCAN database.^18^ Geospatial modeling was used to estimate population-weighted cancer incidence by region.

Radiotherapy facilities and the number of available machines were identified from the International Atomic Energy Association Directory of Radiotherapy Centres, which is available online as an open-access data source.^19^ This information was verified with Ghanaian experts and clinical staff at each of the 3 radiation treatment facilities. In addition, a retrospective review of radiation treatment records was performed from January 1 to December 31, 2020, at each of the 3 facilities. Demographic data were used to geocode all patients receiving radiotherapy in Ghana during the study period.

### Statistical Analysis

#### Radiotherapy Utilization

The actual radiotherapy utilization rates (ARURs) at the national and regional levels were estimated as the number of new patients treated with at least 1 course of radiotherapy divided by the number of estimated incident cancer cases in 2020. Cancer incidence in each of the 16 regions in Ghana was estimated by weighting the incidence by the population distribution in each region.

The optimal radiotherapy utilization rate (ORUR) was based on the number of incident cancer cases in 2020 using the model of optimum radiotherapy utilization developed by the Australian Collaboration for Cancer Outcomes Research and Evaluation and the Global Task Force on Radiotherapy for Cancer Control (GTFRCC) investment framework.^1,20^ Due to the lack of stage distribution data in Ghana, these estimates relied on the HIC stage distribution used by the GTFRCC. The ORUR was defined as the proportion of new cancer cases with an indication for radiotherapy based on evidence-based guidelines.^20^

#### Radiotherapy Treatment Capacity

The number of radiotherapy machines needed to treat all patients with an indication for radiotherapy was calculated using the GTFRCC’s Radiotherapy Resources and Cost Calculator, which has been used in several studies of radiotherapy resource estimates in LMICs.^1,21^ Radiotherapy facilities were assumed to operate 12 hours per day, 5 days per week, 50 weeks per year, which is consistent with the average operating parameters of the 3 facilities in Ghana.^21,22^ These assumptions were derived through questionnaires to GTFRCC members and consultations by the International Atomic Energy Agency with international facilities of varying size, academic affiliation, and location, and public databases.^1^ These estimates were also reviewed by the Ghanaian authors (A.A.S., C.A.-A., C.E., B.D., J.Y., and E.O.-B.) of this article to verify the validity of these estimates in the Ghanaian context.

We assumed that patients were treated with an average of 19.4 fractions per EBRT course and 3 brachytherapy fractions per course for cervical cancer, based on the model.^21,22^ Brachytherapy was assumed to be an essential component of treatment for cervical cancer but not for other disease sites. We then estimated the gap between the number of EBRT and brachytherapy fractions that can be delivered based on current capacity and the number of fractions required to meet the optimal utilization target.

#### Geospatial Analysis

Geographic accessibility was calculated as the Euclidean distances from Ghana’s district centroids to the nearest radiotherapy facility, which was estimated for each of the 260 districts. The median Euclidean distance was also calculated for Ghana’s 16 regions. A Pearson correlation coefficient was computed to assess the linear association between the log-transformed values for ARUR and the distance to the nearest RT center. *P* < .05 was considered significant.

We also estimated the proportion of the population living approximately 100, 150, and 200 km radii from each radiotherapy facility, which were termed buffer zones. These data were estimated using the gridded population distribution data from WorldPop,^17,23^ which relies on satellite imagery and a machine-learning random forest algorithm to produce its estimates.^24^

Finally, to determine the outcome of adding a new radiotherapy facility to improve geographic accessibility of the population to radiotherapy, we modeled a hypothetical new facility located in Tamale, the northern region’s capital. This location was selected because it is currently undergoing expansion of other cancer services, including surgery, pathology, and imaging, which would enable integration of radiotherapy as a key component of the cancer care continuum. This new center could alleviate the lack of any radiotherapy infrastructure in the northern half of the country. We used QGIS version 2.18 (QGIS Association) for the geospatial analysis and JMP version 14 (SAS Institute) for all other statistical analyses.

## Results

**Figure 1.**
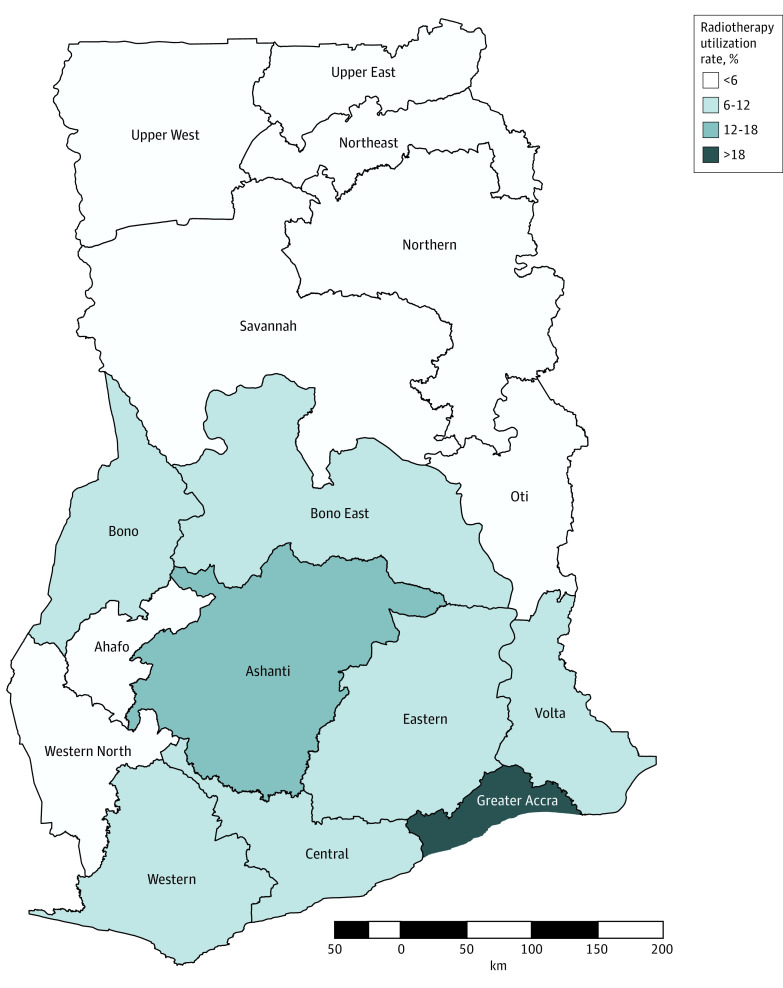
Actual Radiotherapy Utilization Rate by Region

A retrospective review of radiation records from the 3 radiotherapy facilities in Ghana determined that 2883 patients received radiotherapy in 2020, which included 55 930 EBRT fractions and 546 brachytherapy fractions. Of the total radiotherapy courses delivered, 2708 (94%) were delivered to Ghanaian nationals, 101 (4%) to foreign nationals, and 35 (1%) to patients of unknown origin. Radiotherapy demand and utilization by region are shown in Table 1. Based on the 2020 cancer incidence of 24 009 individuals and the number of delivered courses to the Ghanaian population (2708), the ARUR was estimated at 11%. The ARUR was highest in the Greater Accra region (30%) and lowest in the northeast region (1%), which is farthest from the available radiotherapy facilities (Figure 1).

**Table 1.** Cancer Incidence and Radiotherapy Demand by Region in Ghana in 2020

Region	Capital	Population (N = 31 489 256)	Cancer incidence (n = 24 341)^a^	RT		ARUR by region, %
				Demand, No. (%) (n = 11684)^b^	Cases treated (n = 2708)^c^	
Ahafo	Goaso	927 960	717	344 (3)	24	3
Ashanti	Kumasi	5 924 498	4580	2198 (19)	621	14
Bono	Sunyani	1 168 807	904	434 (4)	105	12
Bono East	Techiman	594 712	460	221 (2)	39	9
Central	Cape Coast	2 605 492	2014	967 (8)	185	9
Eastern	Koforidua	3 318 853	2566	1231 (11)	186	7
Greater Accra	Accra	5 055 883	3908	1876 (16)	1168	30
North East	Nalerigu	588 800	455	218 (2)	3	1
Northern	Tamale	1 948 913	1507	723 (6)	54	4
Oti	Dambai	759 799	587	282 (2)	14	2
Savannah	Damongo	1 133 768	876	421 (4)	13	2
Upper East	Bolgatanga	1 302 718	1007	483 (4)	40	4
Upper West	Wa	868 479	671	322 (3)	25	4
Volta	Ho	1 907 679	1475	708 (6)	97	7
Western	Sekondi-Takoradi	2 214 660	1712	822 (7)	109	6
Western North	Sefwi Wiaso	1 168 235	903	433 (4)	25	3

Abbreviations: ARUR, actual radiotherapy utilization rate; RT, radiotherapy.

^a^ Weighted based on population distribution (crude incidence rate of 77.3 cases per 100 000 population). This weighting method and the slight uncertainties in the number of cases by region account for a discrepancy of 332 cases between the sum of the regional totals and national incidence estimated by GLOBOCAN.

^b^ Number of patients requiring RT, assuming an optimal RT utilization rate of 48%.

^c^ Number of patients receiving RT in Ghana; treatment to 101 foreign nationals and 35 patients whose address was unknown were excluded.

Applying the ORUR model to the overall cancer incidence and stage mix, 49% of patients (11 684) in Ghana required radiotherapy treatment in 2020. This indicated that 8976 patients (77%) with an evidence-based indication for radiotherapy did not receive treatment. The highest demand for radiotherapy, based on EBRT requirements, was in the Ashanti region (19%) and the lowest demand was in the Bono East, Northeast, and Oti regions (all 2%) (Table 1). Meeting this demand would require a total of 223 572 EBRT fractions (11 524 EBRT courses) and 5035 brachytherapy fractions (1678 courses) for cervical cancer for optimal utilization. This represents a deficit of 167 642 EBRT fractions (8641 courses) and 4489 brachytherapy fractions. Based on the modeled operating parameter assumptions, Ghana would need to invest in a total of 23 MV units and 4 high-dose rate (HDR) afterloaders to meet the total demand.

**Figure 2.**
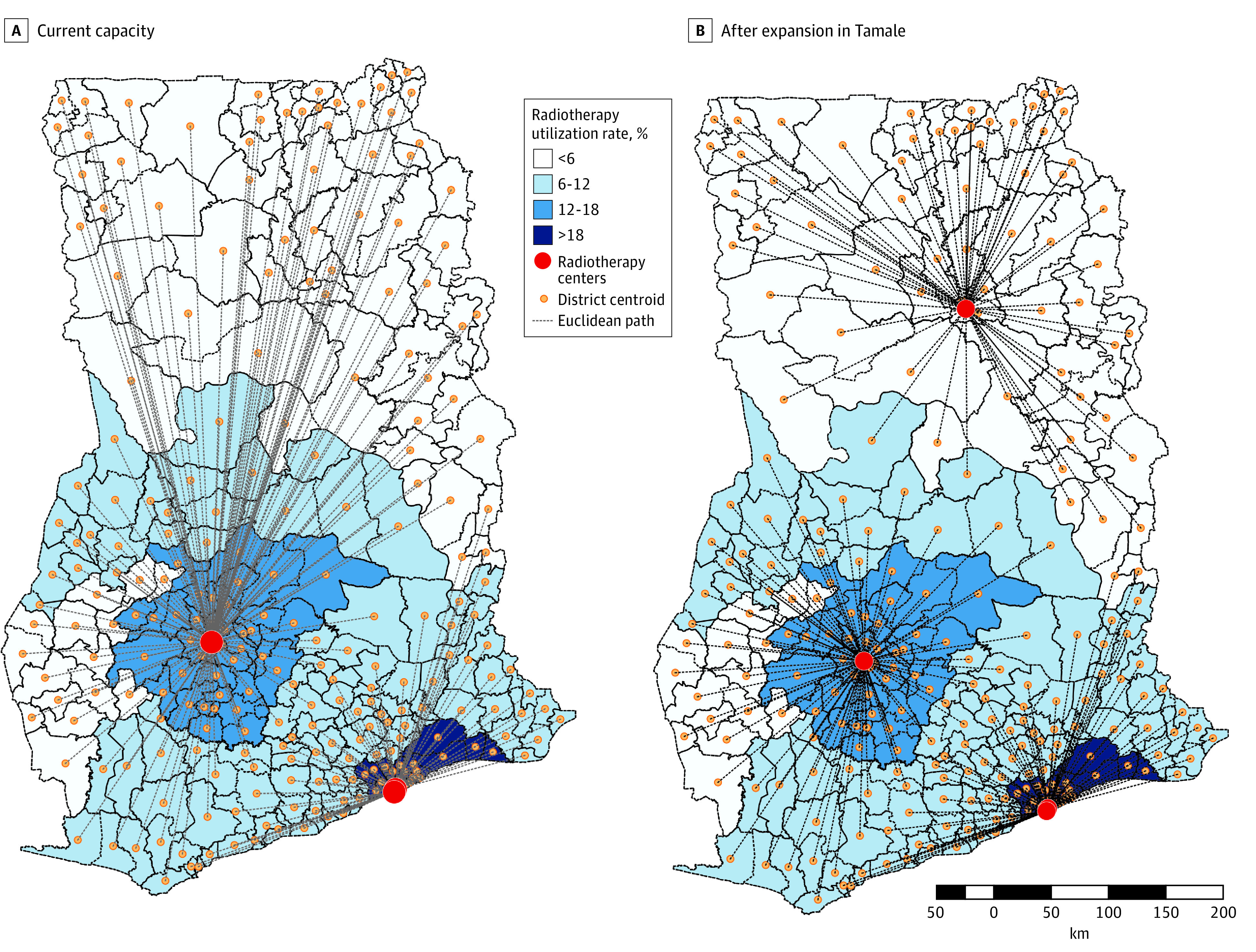
Euclidean Distance From District Centroids to Nearest Radiotherapy Facility The reference radiotherapy facility is the facility that is closest to the capital. There are 3 radiotherapy centers available for the entire country, with 1 center located in Kumasi (Komfo Anokye Teaching Hospital) and 2 centers in Accra (Swedish Ghana Medical Centre and Korle Bu Teaching Hospital). In panel B, a proposed fourth center in Tamale is included. The Euclidean distances from the centroids of the various districts to the nearest radiotherapy facility were calculated. These distances were then aggregated by region, obtaining an estimate of the median Euclidean distance from district centroid to the nearest radiotherapy facility for each of the 16 regions.

The median Euclidean distance from the district centroids to the nearest radiotherapy facility was 110.6 km (range, 0.6-513.2 km) (Figure 2A). The longest distance was between the centroid of the Pusiga district in the Upper East region to the Komfo Anokye Teaching Hospital in Kumasi in the Ashanti region (513.2 km). The median Euclidean distance by region is shown in Table 2, and the distances from each of the 260 district centroids to the nearest radiotherapy facility are presented in the eTable in the Supplement. A significant negative correlation was observed between the ARUR and the median regional Euclidean distance to the nearest radiotherapy facility (*r*_(15)_ = 0.66; *P* < .001).

**Table 2.** Euclidean Distance From District Centroid to Nearest Radiotherapy Facility Before and After Expansion of a New Facility in Tamale, by Region

Region	Districts, No.	Current RT capacity		After expansion to Tamale	
		Nearest RT center	Distance from district centroids to nearest RT facility, median (range), km	Nearest RT center	Distance from district centroids to nearest RT facility, median (range), km
Ahafo	6	Kumasi for all districts	93.4 (67.3-117.5)	Kumasi for all districts	93.4 (67.3-117.5)
Ashanti	43	Kumasi for all districts	42.6 (1.3-113.7)	Kumasi for all districts	42.6 (1.3-113.7)
Bono	12	Kumasi for all districts	145.1 (100.8-192.4)	Kumasi for all districts	145.1 (100.8-192.4)
Bono East	11	Kumasi for all districts	139.4 (85.2-188.4)	Kumasi for 9 districts; Tamale for 2 districts	138.0 (85.2-188.4)
Central	22	Accra for 17 districts; Kumasi for 5 districts	88.1 (30.3-144.4)	Accra for 17 districts; Kumasi for 5 districts	88.1 (30.3-144.4)
Eastern	33	Accra for 26 districts; Kumasi for 7 districts	89.6 (33.1-150.8)	Accra for 26 districts; Kumasi for 7 districts	89.6 (33.1-150.8)
Greater Accra	29	Accra for all districts	15.6 (0.6-89.7)	Accra for all districts	15.6 (0.6-89.7)
North East	6	Kumasi for all districts	437.1 (395.3-460.5)	Tamale for all districts	133.1 (102.0-153.6)
Northern	16	Kumasi for all districts	340.2 (259.1-393.2)	Tamale for all districts	83.6 (7.5-142.7)
Oti	8	Kumasi for 5 districts; Accra for 3 districts	237.2 (207.1-293.6)	Tamale for 6 districts; Accra for 2 districts	199.0 (151.5-239.2)
Savannah	7	Kumasi for all districts	257.3 (196.9-333.9)	Tamale for all districts	108.7 (71.6-173.7)
Upper East	15	Kumasi for all districts	471.6 (427.4-513.2)	Tamale for all districts	163.1 (136.9-201.1)
Upper West	11	Kumasi for all districts	439.7 (371.9-478.5)	Tamale for all districts	219.7 (149.6-267.7)
Volta	18	Accra for all districts	135.9 (88.2-193.7)	Accra for all districts	135.9 (88.2-193.7)
Western	14	Kumasi for 11 districts; Accra for 3 districts	178.5 (104.6-208.3)	Kumasi for 11 districts; Accra for 3 districts	178.5(104.6-208.3)
Western North	9	Kumasi for all districts	146.2 (83.4-167.3)	Kumasi for all districts	146.2 (83.4-167.3)

Abbreviation: RT, radiotherapy.

The construction of a new radiotherapy facility in the Northern regional capital of Tamale, where the ARUR is low, would reduce the median distance from the district centroid to the nearest radiotherapy facility by 10%, to 99.4 km (range, 0.6-267.7 km) (Figure 2B). The greatest benefit would be seen in the Savannah, Upper West, Northern, Northeast, and Upper East regions, which are all located in northern Ghana (eFigure in the Supplement).

**Figure 3.**
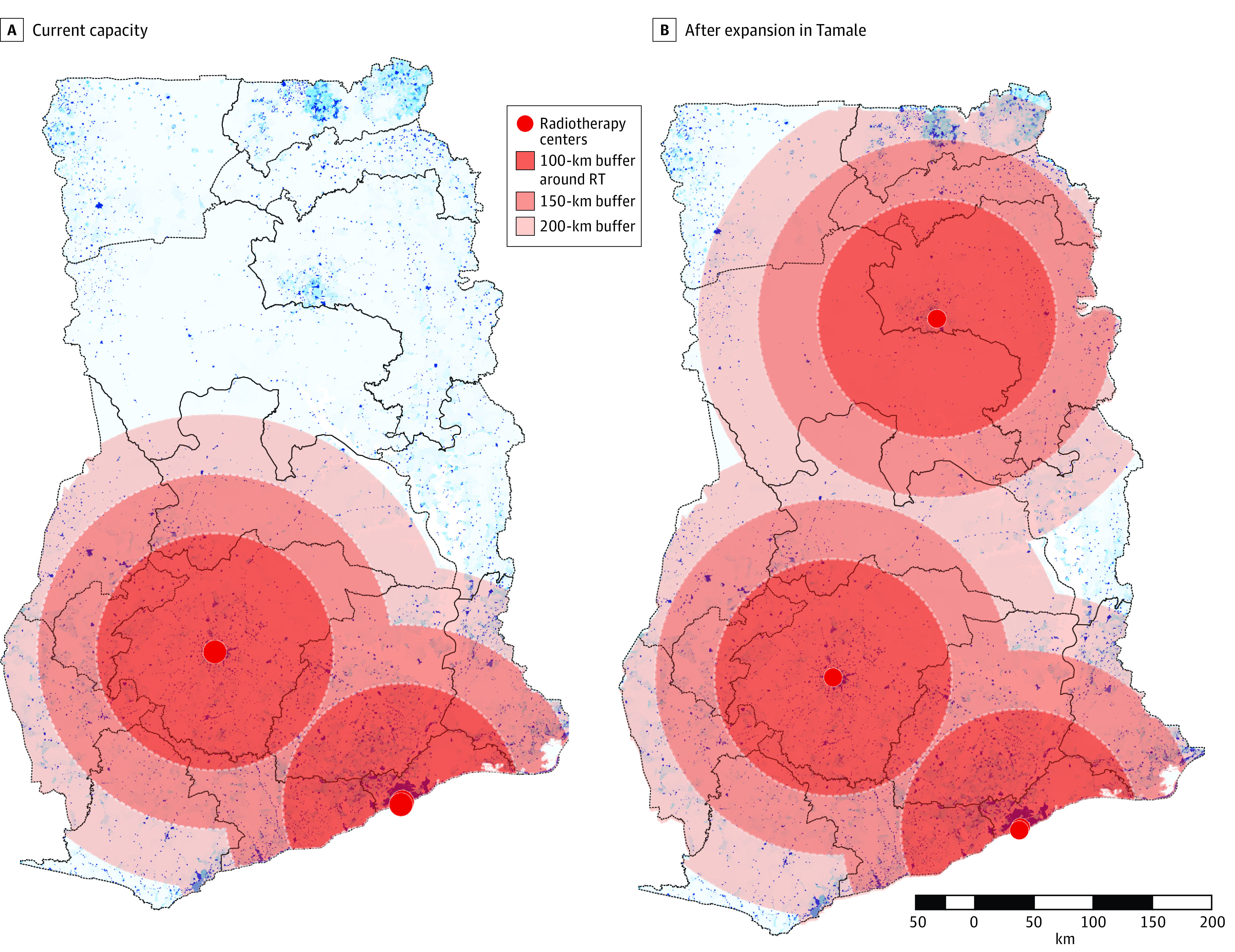
Proportion of the Population Living Within 100-km, 150-km, and 200-km Buffer Zones of a Radiotherapy Facility

Based on current capacity, the proportion of the total population living within a radius of 100, 150, and 200 km of a radiotherapy center was 47%, 61% and 70%, respectively (Figure 3A). The expansion in Tamale would increase the proportion of the population living within the 100, 150, and 200 km buffer zones to 53%, 69%, and 84%, respectively (Figure 3B).

## Discussion

Using national data on radiotherapy delivery and geospatial analytic approaches, we estimated that 74% of cancer patients in Ghana who required radiotherapy in 2020 did not receive it and that the national shortfall in radiotherapy availability was compounded by significant regional disparities in access. We geocoded the region of residence of all patients receiving radiotherapy treatment in Ghana and found that nearly half of patients traveled close to 200 km to access a radiotherapy facility and that regions with longer travel distances to radiotherapy facilities were associated with lower rates of radiotherapy utilization. To our knowledge, this is the first study to describe the patterns of radiotherapy access in Ghana and to propose a solution to inform health system planning in the form of a strategically constructed facility in the north of the country.

Geographic accessibility is particularly critical in settings with a largely rural population such as Ghana, where 43% of the population lives in rural communities.^14^ Available radiotherapy facilities were clustered in 2 cities in the south of Ghana, resulting in a median Euclidean distance of 110 km to access radiotherapy services, and less than half the population living within 100 km of a radiotherapy facility. By contrast, a study of radiotherapy accessibility in Malaysia using road distance found that only 25% of the population traveled more than 100 km to the nearest radiotherapy center.^25^ Similar findings have been observed in access to other health services in Ghana. A study of geographic accessibility of maternity care found that 34% of women in Ghana lived beyond the clinically significant threshold of a 2-hour drive from facilities that are likely to offer emergency obstetric and neonatal care, and this increased to 63% in the most remote regions.^26^

Our findings also demonstrated a strong association between geographic accessibility and radiotherapy utilization. All 3 existing radiotherapy facilities are in southern Ghana, where population density is highest, but this leaves populations in the northern regions without accessible services. This north-south disparity in radiation accessibility was reflected in the ARUR variation observed, in which utilization was less than 6% in the northern half of the country compared with 30% in Greater Accra in the south. Studies across the world have found an association between distance to radiotherapy facilities, utilization, and outcomes from care. A systematic review of distance to a radiotherapy center and utilization of palliative radiotherapy among adults with cancer^27^ found that patients living 50 km or greater from a radiotherapy center were less likely to receive palliative radiotherapy compared with those living less than 50 km away.^27^ In Nigeria, significant variability in access to a comprehensive cancer center between geopolitical zones has been observed,^28^ and geographic accessibility to such tertiary care facilities has been independently associated with stage at presentation and overall survival among patients with breast cancer.^29^ In Brazil, the number of linear accelerators in different regions of the country was directly correlated with the number of patients treated in those regions.^30^

This study’s findings of disparities in geographic access can provide important information to inform policy makers and development partners. In 2008, a National Cancer Control Steering Committee was established in Ghana to advise the government on health system planning, oversee the establishment and operation of a National Cancer Control Programme, and develop the National Strategy for Cancer Control in Ghana.^31,32^ Although there is a clear strategic direction for cancer control, which includes improved access to radiotherapy and other cancer treatment, the program has received criticism for being underresourced to implement its planned activities.^31^ In the setting of limited resources and competing priorities, the data from this study can help guide targeted and equity-focused investment. Our analysis of the radiotherapy shortfall in Ghana found that a total of 23 EBRT machines and 4 HDR brachytherapy afterloaders were needed to meet the current demand for radiotherapy. Although the implementation of 1 additional facility with 2 EBRT machines still leaves an enormous unmet need for treatment, our modeled analysis provides important information to guide an incremental scale-up approach for a new facility in a location that would minimize geographic disparities. Establishing a new radiotherapy center in Tamale, where radiotherapy utilization was among the lowest in the country, could significantly reduce the travel distances for several districts in the country, where radiotherapy is largely inaccessible. This approach can be considered as Ghana and other countries expand their radiotherapy services.

### Limitations

This study must be considered in the context of its limitations. Although Euclidean distances are a common method of evaluating travel impedance in public health research, our analytic approach did not account for other barriers to travel, such as water bodies, land elevations, mode of transportation, and lack of road access. Furthermore, our focus on radiotherapy availability and geographic accessibility did not consider factors such as affordability, referral patterns from primary health facilities, and trust in health care professionals, which may all shape a patient’s ability to seek care. Nevertheless, the estimates in the present study offer a reproducible method to compare with similar countries in the region.

In addition, our estimates of regional cancer incidence relied on national estimates from GLOBOCAN, which were weighted in proportion to the population living in each region. The precise estimates of the population within each region, the age and sex distribution by region, as well as the distribution of cases by cancer stage were not readily available from the Ghana Statistical Service, which may affect our estimates of ORUR and the gap in radiotherapy. However, a sensitivity analysis performed by the GTFRCC to determine the how more advanced stages of cancer would affect estimates of ORUR, which are typically present in LMICs such as Ghana, found that this resulted in only a 4% overall increase in the ORUR. As a result, our estimates of radiotherapy demand are likely a conservative estimate of radiotherapy demand.

## Conclusions

In this cross-sectional study of the geographic accessibility and availability of radiotherapy, Ghana was found to have a significant shortage of radiotherapy facilities to meet the needs of its population and large regional disparities in access to existing services. The data from this study on the additional radiotherapy requirements needed to service the population and the benefit of considering spatial accessibility in plans for scale-up can provide important information for health system planning. Improved understanding of regional disparities in access is an important finding for international efforts to improve radiotherapy access.

## Notes

Supplement. **eTable.** Euclidean Distances From District Centroids to Nearest Radiotherapy Facility **eFigure.** Distance From District Centroid to Nearest Radiotherapy Facility
