# Supplementary material for: Geographic Accessibility and Availability of Radiotherapy in Ghana
Source: JAMA Netw Open. 2022 Aug 11;5(8):e2226319. doi: 10.1001/jamanetworkopen.2022.26319 (PMC9372791; doi:10.1001/jamanetworkopen.2022.26319)
Supplement: Supplement. — eTable. Euclidean Distances From District Centroids to Nearest Radiotherapy Facility eFigure. Distance From District Centroid to Nearest Radiotherapy Facility [file jamanetwopen-e2226319-s001.pdf]

Supplemental Online Content

Scott AA, Polo A, Zubizarreta E, et al. Geographic accessibility and availability of radiotherapy in Ghana. *JAMA Netw Open.* 2022;5(8):e2226319. doi:10.1001/jamanetworkopen.2022.26319

eTable. Euclidean Distances From District Centroids to Nearest Radiotherapy Facility

eFigure. Distance From District Centroid to Nearest Radiotherapy Facility

This supplemental material has been provided by the authors to give readers additional information about their work.

© 2022 Scott AA et al. *JAMA Network Open.*

eTable. Euclidean Distances From District Centroids to Nearest Radiotherapy Facility

Region	District name	Radiotherapy facility before expansion	Euclidean Distance (kilometers)	Radiotherapy facility after expansion	Euclidean Distance (kilometers)
AHAFO	TANO SOUTH MUNICIPAL	Kumasi (Komfo Anokye Teaching Hospital)	67,33	Kumasi (Komfo Anokye Teaching Hospital)	67,33
AHAFO	TANO NORTH MUNICIPAL	Kumasi (Komfo Anokye Teaching Hospital)	81,34	Kumasi (Komfo Anokye Teaching Hospital)	81,34
AHAFO	ASUTIFI SOUTH	Kumasi (Komfo Anokye Teaching Hospital)	85,62	Kumasi (Komfo Anokye Teaching Hospital)	85,62
AHAFO	ASUNAFO SOUTH	Kumasi (Komfo Anokye Teaching Hospital)	101,98	Kumasi (Komfo Anokye Teaching Hospital)	101,98
AHAFO	ASUTIFI NORTH	Kumasi (Komfo Anokye Teaching Hospital)	106,34	Kumasi (Komfo Anokye Teaching Hospital)	106,34
AHAFO	ASUNAFO NORTH MUNICIPAL	Kumasi (Komfo Anokye Teaching Hospital)	117,59	Kumasi (Komfo Anokye Teaching Hospital)	117,59
ASHANTI	KUMASI METROPOLITAN	Kumasi (Komfo Anokye Teaching Hospital)	1,30	Kumasi (Komfo Anokye Teaching Hospital)	1,30
ASHANTI	KWADASO MUNICIPAL	Kumasi (Komfo Anokye Teaching Hospital)	4,30	Kumasi (Komfo Anokye Teaching Hospital)	4,30
ASHANTI	OLD TAFO MUNICIPAL	Kumasi (Komfo Anokye Teaching Hospital)	5,12	Kumasi (Komfo Anokye Teaching Hospital)	5,12
ASHANTI	SUAME MUNICIPAL	Kumasi (Komfo Anokye Teaching Hospital)	5,68	Kumasi (Komfo Anokye Teaching Hospital)	5,68
ASHANTI	ASOKORE MAMPONG MUNICIPAL	Kumasi (Komfo Anokye Teaching Hospital)	6,37	Kumasi (Komfo Anokye Teaching Hospital)	6,37
ASHANTI	ASOKWA MUNICIPAL	Kumasi (Komfo Anokye Teaching Hospital)	7,40	Kumasi (Komfo Anokye Teaching Hospital)	7,40
ASHANTI	OFORIKROM MUNICIPAL	Kumasi (Komfo Anokye Teaching Hospital)	9,28	Kumasi (Komfo Anokye Teaching Hospital)	9,28
ASHANTI	ATWIMA KWANWOMA	Kumasi (Komfo Anokye Teaching Hospital)	14,59	Kumasi (Komfo Anokye Teaching Hospital)	14,59
ASHANTI	KWABRE EAST	Kumasi (Komfo Anokye Teaching Hospital)	15,22	Kumasi (Komfo Anokye Teaching Hospital)	15,22
ASHANTI	AFIGYA KWABRE SOUTH	Kumasi (Komfo Anokye Teaching Hospital)	16,53	Kumasi (Komfo Anokye Teaching Hospital)	16,53
ASHANTI	ATWIMA NWABIAGYA SOUTH	Kumasi (Komfo Anokye Teaching Hospital)	18,13	Kumasi (Komfo Anokye Teaching Hospital)	18,13
ASHANTI	ATWIMA NWABIAGYA NORTH	Kumasi (Komfo Anokye Teaching Hospital)	23,01	Kumasi (Komfo Anokye Teaching Hospital)	23,01
ASHANTI	BOSOMTWE	Kumasi (Komfo Anokye Teaching Hospital)	24,24	Kumasi (Komfo Anokye Teaching Hospital)	24,24
ASHANTI	EJISU MUNICIPAL	Kumasi (Komfo Anokye Teaching Hospital)	25,80	Kumasi (Komfo Anokye Teaching Hospital)	25,80
ASHANTI	JUABEN MUNICIPAL	Kumasi (Komfo Anokye Teaching Hospital)	28,27	Kumasi (Komfo Anokye Teaching Hospital)	28,27
ASHANTI	BEKWAI MUNICIPAL	Kumasi (Komfo Anokye Teaching Hospital)	28,81	Kumasi (Komfo Anokye Teaching Hospital)	28,81

© 2022 Scott AA et al. *JAMA Network Open.*

ASHANTI	SEKYERE SOUTH	Kumasi (Komfo Anokye Teaching Hospital)	34,40	Kumasi (Komfo Anokye Teaching Hospital)	34,40
**Region**	**District name**	**Radiotherapy facility before expansion**	**Euclidean Distance_1**	**Radiotherapy facility after expansion**	**Euclidean Distance_2**
ASHANTI	SEKYERE EAST	Kumasi (Komfo Anokye Teaching Hospital)	35,38	Kumasi (Komfo Anokye Teaching Hospital)	35,38
ASHANTI	AMANSIE WEST	Kumasi (Komfo Anokye Teaching Hospital)	36,34	Kumasi (Komfo Anokye Teaching Hospital)	36,34
ASHANTI	AFIGYA KWABRE NORTH	Kumasi (Komfo Anokye Teaching Hospital)	37,90	Kumasi (Komfo Anokye Teaching Hospital)	37,90
ASHANTI	AHAFO ANO SOUTH EAST	Kumasi (Komfo Anokye Teaching Hospital)	38,13	Kumasi (Komfo Anokye Teaching Hospital)	38,13
ASHANTI	OFFINSO MUNICIPAL	Kumasi (Komfo Anokye Teaching Hospital)	42,58	Kumasi (Komfo Anokye Teaching Hospital)	42,58
ASHANTI	ASANTE AKIM CENTRAL MUNICIPAL	Kumasi (Komfo Anokye Teaching Hospital)	45,73	Kumasi (Komfo Anokye Teaching Hospital)	45,73
ASHANTI	ADANSI NORTH	Kumasi (Komfo Anokye Teaching Hospital)	45,82	Kumasi (Komfo Anokye Teaching Hospital)	45,82
ASHANTI	SEKYERE KUMAWU	Kumasi (Komfo Anokye Teaching Hospital)	50,46	Kumasi (Komfo Anokye Teaching Hospital)	50,46
ASHANTI	BOSOME FREHO	Kumasi (Komfo Anokye Teaching Hospital)	53,13	Kumasi (Komfo Anokye Teaching Hospital)	53,13
ASHANTI	AHAFO ANO SOUTH WEST	Kumasi (Komfo Anokye Teaching Hospital)	53,91	Kumasi (Komfo Anokye Teaching Hospital)	53,91
ASHANTI	OBUASI MUNICIPAL	Kumasi (Komfo Anokye Teaching Hospital)	55,95	Kumasi (Komfo Anokye Teaching Hospital)	55,95
ASHANTI	MAMPONG MUNICIPAL	Kumasi (Komfo Anokye Teaching Hospital)	56,09	Kumasi (Komfo Anokye Teaching Hospital)	56,09
ASHANTI	AMANSIE CENTRAL	Kumasi (Komfo Anokye Teaching Hospital)	56,68	Kumasi (Komfo Anokye Teaching Hospital)	56,68
ASHANTI	OBUASI EAST	Kumasi (Komfo Anokye Teaching Hospital)	57,08	Kumasi (Komfo Anokye Teaching Hospital)	57,08
ASHANTI	ATWIMA MPONUA	Kumasi (Komfo Anokye Teaching Hospital)	58,82	Kumasi (Komfo Anokye Teaching Hospital)	58,82
ASHANTI	AMANSIE SOUTH	Kumasi (Komfo Anokye Teaching Hospital)	59,87	Kumasi (Komfo Anokye Teaching Hospital)	59,87
ASHANTI	ASANTE AKIM SOUTH	Kumasi (Komfo Anokye Teaching Hospital)	61,38	Kumasi (Komfo Anokye Teaching Hospital)	61,38
ASHANTI	ADANSI ASOKWA	Kumasi (Komfo Anokye Teaching Hospital)	61,80	Kumasi (Komfo Anokye Teaching Hospital)	61,80
ASHANTI	AHAFO ANO NORTH	Kumasi (Komfo Anokye Teaching Hospital)	69,07	Kumasi (Komfo Anokye Teaching Hospital)	69,07
ASHANTI	ASANTE AKIM NORTH	Kumasi (Komfo Anokye Teaching Hospital)	72,42	Kumasi (Komfo Anokye Teaching Hospital)	72,42
ASHANTI	OFFINSO NORTH	Kumasi (Komfo Anokye Teaching Hospital)	73,21	Kumasi (Komfo Anokye Teaching Hospital)	73,21
ASHANTI	ADANSI AKROFUOM	Kumasi (Komfo Anokye Teaching Hospital)	73,96	Kumasi (Komfo Anokye Teaching Hospital)	73,96

© 2022 Scott AA et al. *JAMA Network Open.*

ASHANTI	SEKYERE CENTRAL	Kumasi (Komfo Anokye Teaching Hospital)	78,58	Kumasi (Komfo Anokye Teaching Hospital)	78,58
**Region**	**District name**	**Radiotherapy facility before expansion**	**Euclidean Distance_1**	**Radiotherapy facility after expansion**	**Euclidean Distance_2**
ASHANTI	EJURA-SEKYEDUMASE	Kumasi (Komfo Anokye Teaching Hospital)	80,52	Kumasi (Komfo Anokye Teaching Hospital)	80,52
ASHANTI	ADANSI SOUTH	Kumasi (Komfo Anokye Teaching Hospital)	81,64	Kumasi (Komfo Anokye Teaching Hospital)	81,64
ASHANTI	SEKYERE AFRAM PLAINS NORTH	Kumasi (Komfo Anokye Teaching Hospital)	113,79	Kumasi (Komfo Anokye Teaching Hospital)	113,79
BONO	SUNYANI MUNICIPAL	Kumasi (Komfo Anokye Teaching Hospital)	100,81	Kumasi (Komfo Anokye Teaching Hospital)	100,81
BONO	SUNYANI WEST	Kumasi (Komfo Anokye Teaching Hospital)	114,52	Kumasi (Komfo Anokye Teaching Hospital)	114,52
BONO	DORMAA EAST	Kumasi (Komfo Anokye Teaching Hospital)	130,95	Kumasi (Komfo Anokye Teaching Hospital)	130,95
BONO	WENCHI MUNICIPAL	Kumasi (Komfo Anokye Teaching Hospital)	131,64	Kumasi (Komfo Anokye Teaching Hospital)	131,64
BONO	BEREKUM EAST MUNICIPAL	Kumasi (Komfo Anokye Teaching Hospital)	138,72	Kumasi (Komfo Anokye Teaching Hospital)	138,72
BONO	BEREKUM WEST	Kumasi (Komfo Anokye Teaching Hospital)	143,20	Kumasi (Komfo Anokye Teaching Hospital)	143,20
BONO	TAIN	Kumasi (Komfo Anokye Teaching Hospital)	147,08	Kumasi (Komfo Anokye Teaching Hospital)	147,08
BONO	DORMAA MUNICIPAL	Kumasi (Komfo Anokye Teaching Hospital)	148,22	Kumasi (Komfo Anokye Teaching Hospital)	148,22
BONO	DORMAA WEST	Kumasi (Komfo Anokye Teaching Hospital)	150,92	Kumasi (Komfo Anokye Teaching Hospital)	150,92
BONO	JAMAN SOUTH MUNICIPAL	Kumasi (Komfo Anokye Teaching Hospital)	165,85	Kumasi (Komfo Anokye Teaching Hospital)	165,85
BONO	JAMAN NORTH	Kumasi (Komfo Anokye Teaching Hospital)	176,06	Kumasi (Komfo Anokye Teaching Hospital)	176,06
BONO	BANDA	Kumasi (Komfo Anokye Teaching Hospital)	192,40	Kumasi (Komfo Anokye Teaching Hospital)	192,40
BONO EAST	NKORANZA SOUTH	Kumasi (Komfo Anokye Teaching Hospital)	85,30	Kumasi (Komfo Anokye Teaching Hospital)	85,30
BONO EAST	TECHIMAN MUNICIPAL	Kumasi (Komfo Anokye Teaching Hospital)	99,97	Kumasi (Komfo Anokye Teaching Hospital)	99,97
BONO EAST	NKORANZA NORTH	Kumasi (Komfo Anokye Teaching Hospital)	111,41	Kumasi (Komfo Anokye Teaching Hospital)	111,41
BONO EAST	TECHIMAN NORTH	Kumasi (Komfo Anokye Teaching Hospital)	116,89	Kumasi (Komfo Anokye Teaching Hospital)	116,89
BONO EAST	ATEBUBU AMANTIN	Kumasi (Komfo Anokye Teaching Hospital)	128,91	Kumasi (Komfo Anokye Teaching Hospital)	128,91
BONO EAST	KINTAMPO SOUTH	Kumasi (Komfo Anokye Teaching Hospital)	139,42	Kumasi (Komfo Anokye Teaching Hospital)	139,42
BONO EAST	PRU WEST	Kumasi (Komfo Anokye Teaching Hospital)	148,84	Kumasi (Komfo Anokye Teaching Hospital)	148,84

© 2022 Scott AA et al. *JAMA Network Open.*

BONO EAST	SENE WEST	Kumasi (Komfo Anokye Teaching Hospital)	163,48	Kumasi (Komfo Anokye Teaching Hospital)	163,48
**Region**	**District name**	**Radiotherapy facility before expansion**	**Euclidean Distance_1**	**Radiotherapy facility after expansion**	**Euclidean Distance_2**
BONO EAST	PRU EAST	Kumasi (Komfo Anokye Teaching Hospital)	182,59	Tamale Teaching Hospital	140,91
BONO EAST	SENE EAST	Kumasi (Komfo Anokye Teaching Hospital)	188,32	Kumasi (Komfo Anokye Teaching Hospital)	188,32
BONO EAST	KINTAMPO NORTH MUNICIPAL	Kumasi (Komfo Anokye Teaching Hospital)	188,49	Tamale Teaching Hospital	138,07
CENTRAL	AWUTU SENYA EAST	Accra (Korle Bu Teaching Hospital)	30,33	Accra (Korle Bu Teaching Hospital)	30,33
CENTRAL	AWUTU SENYA	Accra (Korle Bu Teaching Hospital)	37,29	Accra (Korle Bu Teaching Hospital)	37,29
CENTRAL	GOMOA EAST	Accra (Korle Bu Teaching Hospital)	43,93	Accra (Korle Bu Teaching Hospital)	43,93
CENTRAL	EFFUTU MUNICIPAL	Accra (Korle Bu Teaching Hospital)	47,35	Accra (Korle Bu Teaching Hospital)	47,35
CENTRAL	AGONA EAST	Accra (Korle Bu Teaching Hospital)	51,12	Accra (Korle Bu Teaching Hospital)	51,12
CENTRAL	GOMOA CENTRAL	Accra (Korle Bu Teaching Hospital)	60,54	Accra (Korle Bu Teaching Hospital)	60,54
CENTRAL	AGONA WEST MUNICIPAL	Accra (Korle Bu Teaching Hospital)	65,58	Accra (Korle Bu Teaching Hospital)	65,58
CENTRAL	GOMOA WEST	Accra (Korle Bu Teaching Hospital)	69,45	Accra (Korle Bu Teaching Hospital)	69,45
CENTRAL	UPPER DENKYIRA WEST	Kumasi (Komfo Anokye Teaching Hospital)	78,64	Kumasi (Komfo Anokye Teaching Hospital)	78,64
CENTRAL	EKUMFI	Accra (Korle Bu Teaching Hospital)	83,37	Accra (Korle Bu Teaching Hospital)	83,37
CENTRAL	AJUMAKO-ENYAN-ESSIAM	Accra (Korle Bu Teaching Hospital)	87,09	Accra (Korle Bu Teaching Hospital)	87,09
CENTRAL	ASIKUMA-ODOBEN-BRAKWA	Accra (Korle Bu Teaching Hospital)	89,28	Accra (Korle Bu Teaching Hospital)	89,28
CENTRAL	UPPER DENKYIRA EAST MUNICIPAL	Kumasi (Komfo Anokye Teaching Hospital)	93,45	Kumasi (Komfo Anokye Teaching Hospital)	93,45
CENTRAL	ASSIN NORTH	Kumasi (Komfo Anokye Teaching Hospital)	99,28	Kumasi (Komfo Anokye Teaching Hospital)	99,28
CENTRAL	MFANTSEMAN MUNICIPAL	Accra (Korle Bu Teaching Hospital)	104,80	Accra (Korle Bu Teaching Hospital)	104,80
CENTRAL	ASSIN FOSU	Kumasi (Komfo Anokye Teaching Hospital)	113,11	Kumasi (Komfo Anokye Teaching Hospital)	113,11
CENTRAL	TWIFO ATTI-MORKWA	Kumasi (Komfo Anokye Teaching Hospital)	113,44	Kumasi (Komfo Anokye Teaching Hospital)	113,44
CENTRAL	ABURA-ASEBU-KWAMANKESE	Accra (Korle Bu Teaching Hospital)	113,76	Accra (Korle Bu Teaching Hospital)	113,76
CENTRAL	ASSIN SOUTH	Accra (Korle Bu Teaching Hospital)	118,43	Accra (Korle Bu Teaching Hospital)	118,43

CENTRAL	CAPE COAST METROPOLITAN	Accra (Korle Bu Teaching Hospital)	127,06	Accra (Korle Bu Teaching Hospital)	127,06
**Region**	**District name**	**Radiotherapy facility before expansion**	**Euclidean Distance_1**	**Radiotherapy facility after expansion**	**Euclidean Distance_2**
CENTRAL	TWIFO HEMANG LOWER DENKYIRA	Accra (Korle Bu Teaching Hospital)	137,62	Accra (Korle Bu Teaching Hospital)	137,62
CENTRAL	KOMENDA-EDINA-EGUAFO-ABIREM MUNICIPAL	Accra (Korle Bu Teaching Hospital)	144,91	Accra (Korle Bu Teaching Hospital)	144,91
EASTERN	AKWAPEM SOUTH	Accra (Sweden Ghana Medical Centre)	33,20	Accra (Sweden Ghana Medical Centre)	33,20
EASTERN	NSAWAM ADOAGYIRI	Accra (Sweden Ghana Medical Centre)	33,83	Accra (Sweden Ghana Medical Centre)	33,83
EASTERN	AKWAPEM NORTH	Accra (Sweden Ghana Medical Centre)	44,12	Accra (Sweden Ghana Medical Centre)	44,12
EASTERN	UPPER WEST AKIM	Accra (Sweden Ghana Medical Centre)	47,97	Accra (Sweden Ghana Medical Centre)	47,97
EASTERN	AYENSUANO	Accra (Sweden Ghana Medical Centre)	52,43	Accra (Sweden Ghana Medical Centre)	52,43
EASTERN	OKERE	Accra (Sweden Ghana Medical Centre)	55,29	Accra (Sweden Ghana Medical Centre)	55,29
EASTERN	NEW JUABEN SOUTH MUNICIPAL	Accra (Sweden Ghana Medical Centre)	57,93	Accra (Sweden Ghana Medical Centre)	57,93
EASTERN	SUHUM MUNICIPAL	Accra (Sweden Ghana Medical Centre)	58,12	Accra (Sweden Ghana Medical Centre)	58,12
EASTERN	NEW JUABEN NORTH MUNICIPAL	Accra (Sweden Ghana Medical Centre)	65,58	Accra (Sweden Ghana Medical Centre)	65,58
EASTERN	WEST AKIM	Accra (Sweden Ghana Medical Centre)	67,50	Accra (Sweden Ghana Medical Centre)	67,50
EASTERN	YILO KROBO	Accra (Sweden Ghana Medical Centre)	67,90	Accra (Sweden Ghana Medical Centre)	67,90
EASTERN	LOWER MANYA	Accra (Sweden Ghana Medical Centre)	73,68	Accra (Sweden Ghana Medical Centre)	73,68
EASTERN	ABUAKWA SOUTH	Accra (Sweden Ghana Medical Centre)	74,13	Accra (Sweden Ghana Medical Centre)	74,13
EASTERN	ASENE AKROSO MANSO	Accra (Korle Bu Teaching Hospital)	75,51	Accra (Korle Bu Teaching Hospital)	75,51
EASTERN	ABUAKWA NORTH	Accra (Sweden Ghana Medical Centre)	78,14	Accra (Sweden Ghana Medical Centre)	78,14
EASTERN	BIRIM NORTH	Kumasi (Komfo Anokye Teaching Hospital)	78,95	Kumasi (Komfo Anokye Teaching Hospital)	78,95
EASTERN	FANTEAKWA SOUTH	Accra (Sweden Ghana Medical Centre)	89,60	Accra (Sweden Ghana Medical Centre)	89,60
EASTERN	AKYEM MANSA	Kumasi (Komfo Anokye Teaching Hospital)	89,85	Kumasi (Komfo Anokye Teaching Hospital)	89,85
EASTERN	DENKYEMBOUR	Accra (Sweden Ghana Medical Centre)	90,01	Accra (Sweden Ghana Medical Centre)	90,01
EASTERN	KWAHU EAST	Kumasi (Komfo Anokye Teaching Hospital)	90,14	Kumasi (Komfo Anokye Teaching Hospital)	90,14

EASTERN	ATIWA WEST	Accra (Sweden Ghana Medical Centre)	90,23	Accra (Sweden Ghana Medical Centre)	90,23
**Region**	**District name**	**Radiotherapy facility before expansion**	**Euclidean Distance_1**	**Radiotherapy facility after expansion**	**Euclidean Distance_2**
EASTERN	UPPER MANYA	Accra (Sweden Ghana Medical Centre)	92,22	Accra (Sweden Ghana Medical Centre)	92,22
EASTERN	ACHIASE	Accra (Korle Bu Teaching Hospital)	92,93	Accra (Korle Bu Teaching Hospital)	92,93
EASTERN	KWAHU WEST	Kumasi (Komfo Anokye Teaching Hospital)	96,18	Kumasi (Komfo Anokye Teaching Hospital)	96,18
EASTERN	ASUOGYAMAN	Accra (Sweden Ghana Medical Centre)	97,89	Accra (Sweden Ghana Medical Centre)	97,89
EASTERN	BIRIM CENTRAL MUNICIPAL	Accra (Sweden Ghana Medical Centre)	99,52	Accra (Sweden Ghana Medical Centre)	99,52
EASTERN	KWAEBIBIREM	Kumasi (Komfo Anokye Teaching Hospital)	101,44	Kumasi (Komfo Anokye Teaching Hospital)	101,44
EASTERN	BIRIM SOUTH	Accra (Korle Bu Teaching Hospital)	103,37	Accra (Korle Bu Teaching Hospital)	103,37
EASTERN	FANTEAKWA NORTH	Accra (Sweden Ghana Medical Centre)	107,06	Accra (Sweden Ghana Medical Centre)	107,06
EASTERN	ATIWA EAST	Accra (Sweden Ghana Medical Centre)	108,38	Accra (Sweden Ghana Medical Centre)	108,38
EASTERN	KWAHU SOUTH	Kumasi (Komfo Anokye Teaching Hospital)	117,15	Kumasi (Komfo Anokye Teaching Hospital)	117,15
EASTERN	KWAHU AFRAM PLAINS SOUTH	Kumasi (Komfo Anokye Teaching Hospital)	141,39	Kumasi (Komfo Anokye Teaching Hospital)	141,39
EASTERN	KWAHU AFRAM PLAINS NORTH	Accra (Sweden Ghana Medical Centre)	150,90	Accra (Sweden Ghana Medical Centre)	150,90
GREATER ACCRA	KORLE KLOTTEY MUNICIPAL	Accra (Sweden Ghana Medical Centre)	0,62	Accra (Sweden Ghana Medical Centre)	0,62
GREATER ACCRA	ACCRA METROPOLIS	Accra (Korle Bu Teaching Hospital)	0,72	Accra (Korle Bu Teaching Hospital)	0,72
GREATER ACCRA	AYAWASO CENTRAL MUNICIPAL	Accra (Sweden Ghana Medical Centre)	2,22	Accra (Sweden Ghana Medical Centre)	2,22
GREATER ACCRA	AYAWASO EAST MUNICIPAL	Accra (Sweden Ghana Medical Centre)	3,54	Accra (Sweden Ghana Medical Centre)	3,54
GREATER ACCRA	ABLEKUMA CENTRAL MUNICIPAL	Accra (Korle Bu Teaching Hospital)	3,63	Accra (Korle Bu Teaching Hospital)	3,63
GREATER ACCRA	AYAWASO NORTH MUNICIPAL	Accra (Sweden Ghana Medical Centre)	4,41	Accra (Sweden Ghana Medical Centre)	4,41
GREATER ACCRA	ABLEKUMA WEST MUNICIPAL	Accra (Korle Bu Teaching Hospital)	4,89	Accra (Korle Bu Teaching Hospital)	4,89
GREATER ACCRA	LA DADE-KOTOPON	Accra (Sweden Ghana Medical Centre)	5,41	Accra (Sweden Ghana Medical Centre)	5,41
GREATER ACCRA	ABLEKUMA NORTH MUNICIPAL	Accra (Sweden Ghana Medical Centre)	5,86	Accra (Sweden Ghana Medical Centre)	5,86
GREATER ACCRA	OKAIKWEI NORTH MUNICIPAL	Accra (Sweden Ghana Medical Centre)	7,35	Accra (Sweden Ghana Medical Centre)	7,35

© 2022 Scott AA et al. *JAMA Network Open.*

GREATER ACCRA	AYAWASO WEST	Accra (Sweden Ghana Medical Centre)	8,70	Accra (Sweden Ghana Medical Centre)	8,70
**Region**	**District name**	**Radiotherapy facility before expansion**	**Euclidean Distance_1**	**Radiotherapy facility after expansion**	**Euclidean Distance_2**
GREATER ACCRA	LEDZOKUKU MUNICIPAL	Accra (Sweden Ghana Medical Centre)	10,27	Accra (Sweden Ghana Medical Centre)	10,27
GREATER ACCRA	GA CENTRAL MUNICIPAL	Accra (Sweden Ghana Medical Centre)	13,54	Accra (Sweden Ghana Medical Centre)	13,54
GREATER ACCRA	KROWOR MUNICIPAL	Accra (Sweden Ghana Medical Centre)	13,79	Accra (Sweden Ghana Medical Centre)	13,79
GREATER ACCRA	GA EAST	Accra (Sweden Ghana Medical Centre)	15,64	Accra (Sweden Ghana Medical Centre)	15,64
GREATER ACCRA	WEIJA GBAWE MUNICIPAL	Accra (Korle Bu Teaching Hospital)	15,69	Accra (Korle Bu Teaching Hospital)	15,69
GREATER ACCRA	ADENTA MUNICIPAL	Accra (Sweden Ghana Medical Centre)	17,22	Accra (Sweden Ghana Medical Centre)	17,22
GREATER ACCRA	TEMA WEST MUNICIPAL	Accra (Sweden Ghana Medical Centre)	18,17	Accra (Sweden Ghana Medical Centre)	18,17
GREATER ACCRA	LA-NKWANTANANG-MADINA	Accra (Sweden Ghana Medical Centre)	20,87	Accra (Sweden Ghana Medical Centre)	20,87
GREATER ACCRA	GA NORTH MUNICIPAL	Accra (Sweden Ghana Medical Centre)	21,24	Accra (Sweden Ghana Medical Centre)	21,24
GREATER ACCRA	TEMA METROPOLITAN	Accra (Sweden Ghana Medical Centre)	23,09	Accra (Sweden Ghana Medical Centre)	23,09
GREATER ACCRA	ASHAIMAN MUNICIPAL	Accra (Sweden Ghana Medical Centre)	24,12	Accra (Sweden Ghana Medical Centre)	24,12
GREATER ACCRA	GA WEST MUNICIPAL	Accra (Sweden Ghana Medical Centre)	25,19	Accra (Sweden Ghana Medical Centre)	25,19
GREATER ACCRA	KPONE KATAMANSO	Accra (Sweden Ghana Medical Centre)	26,82	Accra (Sweden Ghana Medical Centre)	26,82
GREATER ACCRA	GA SOUTH MUNICIPAL	Accra (Sweden Ghana Medical Centre)	29,16	Accra (Sweden Ghana Medical Centre)	29,16
GREATER ACCRA	NINGO/PRAMPRAM	Accra (Sweden Ghana Medical Centre)	49,78	Accra (Sweden Ghana Medical Centre)	49,78
GREATER ACCRA	SHAI OSUDOKU	Accra (Sweden Ghana Medical Centre)	57,62	Accra (Sweden Ghana Medical Centre)	57,62
GREATER ACCRA	ADA WEST	Accra (Sweden Ghana Medical Centre)	77,87	Accra (Sweden Ghana Medical Centre)	77,87
GREATER ACCRA	ADA EAST	Accra (Sweden Ghana Medical Centre)	89,79	Accra (Sweden Ghana Medical Centre)	89,79
NORTHERN	KPANDAI	Kumasi (Komfo Anokye Teaching Hospital)	259,11	Tamale Teaching Hospital	142,73
NORTHERN	NANUMBA SOUTH	Kumasi (Komfo Anokye Teaching Hospital)	292,08	Tamale Teaching Hospital	123,18
NORTHERN	NANUMBA NORTH	Kumasi (Komfo Anokye Teaching Hospital)	298,88	Tamale Teaching Hospital	97,87
NORTHERN	TAMALE METROPOLITAN	Kumasi (Komfo Anokye Teaching Hospital)	312,62	Tamale Teaching Hospital	10,95

© 2022 Scott AA et al. *JAMA Network Open.*

NORTHERN	SAGNERIGU	Kumasi (Komfo Anokye Teaching Hospital)	318,82	Tamale Teaching Hospital	7,59
**Region**	**District name**	**Radiotherapy facility before expansion**	**Euclidean Distance_1**	**Radiotherapy facility after expansion**	**Euclidean Distance_2**
NORTHERN	TOLON	Kumasi (Komfo Anokye Teaching Hospital)	318,98	Tamale Teaching Hospital	40,79
NORTHERN	NANTON	Kumasi (Komfo Anokye Teaching Hospital)	333,50	Tamale Teaching Hospital	22,79
NORTHERN	MION	Kumasi (Komfo Anokye Teaching Hospital)	335,48	Tamale Teaching Hospital	64,55
NORTHERN	ZABZUGU	Kumasi (Komfo Anokye Teaching Hospital)	344,92	Tamale Teaching Hospital	130,91
NORTHERN	KUMBUNGU	Kumasi (Komfo Anokye Teaching Hospital)	348,88	Tamale Teaching Hospital	49,02
NORTHERN	YENDI MUNICIPAL	Kumasi (Komfo Anokye Teaching Hospital)	357,34	Tamale Teaching Hospital	96,93
NORTHERN	TATALE SANGULI	Kumasi (Komfo Anokye Teaching Hospital)	360,22	Tamale Teaching Hospital	140,96
NORTHERN	SAVELUGU	Kumasi (Komfo Anokye Teaching Hospital)	362,04	Tamale Teaching Hospital	50,67
NORTHERN	KARAGA	Kumasi (Komfo Anokye Teaching Hospital)	385,95	Tamale Teaching Hospital	75,06
NORTHERN	GUSHEGU	Kumasi (Komfo Anokye Teaching Hospital)	391,84	Tamale Teaching Hospital	92,27
NORTHERN	SABOBA	Kumasi (Komfo Anokye Teaching Hospital)	393,23	Tamale Teaching Hospital	118,62
NORTHERN EAST	MAMPRUGU MOAGDURI	Kumasi (Komfo Anokye Teaching Hospital)	395,31	Tamale Teaching Hospital	108,21
NORTHERN EAST	WEST MAMPRUSI MUNICIPAL	Kumasi (Komfo Anokye Teaching Hospital)	412,07	Tamale Teaching Hospital	102,06
NORTHERN EAST	CHEREPONI	Kumasi (Komfo Anokye Teaching Hospital)	436,47	Tamale Teaching Hospital	144,75
NORTHERN EAST	EAST MAMPRUSI	Kumasi (Komfo Anokye Teaching Hospital)	437,83	Tamale Teaching Hospital	125,59
NORTHERN EAST	YUNYOO-NASUAN	Kumasi (Komfo Anokye Teaching Hospital)	448,07	Tamale Teaching Hospital	140,80
NORTHERN EAST	BUNKPURUGU NAKPANDURI	Kumasi (Komfo Anokye Teaching Hospital)	460,59	Tamale Teaching Hospital	153,65
OTI	BIAKOYE	Accra (Sweden Ghana Medical Centre)	207,17	Accra (Sweden Ghana Medical Centre)	207,17
OTI	JASIKAN	Accra (Sweden Ghana Medical Centre)	217,28	Accra (Sweden Ghana Medical Centre)	217,28
OTI	KRACHI WEST	Kumasi (Komfo Anokye Teaching Hospital)	223,03	Tamale Teaching Hospital	187,49
OTI	KRACHI NCHUMURU	Kumasi (Komfo Anokye Teaching Hospital)	236,49	Tamale Teaching Hospital	159,70
OTI	KRACHI EAST MUNICIPAL	Kumasi (Komfo Anokye Teaching Hospital)	238,07	Tamale Teaching Hospital	214,25

© 2022 Scott AA et al. *JAMA Network Open.*

OTI	KADJEBI	Accra (Sweden Ghana Medical Centre)	252,85	Tamale Teaching Hospital	239,24
**Region**	**District name**	**Radiotherapy facility before expansion**	**Euclidean Distance_1**	**Radiotherapy facility after expansion**	**Euclidean Distance_2**
OTI	NKWANTA SOUTH MUNICIPAL	Kumasi (Komfo Anokye Teaching Hospital)	288,58	Tamale Teaching Hospital	190,90
OTI	NKWANTA NORTH	Kumasi (Komfo Anokye Teaching Hospital)	293,66	Tamale Teaching Hospital	151,56
SAVANNAH	EAST GONJA MUNICIPAL	Kumasi (Komfo Anokye Teaching Hospital)	196,92	Tamale Teaching Hospital	116,56
SAVANNAH	BOLE	Kumasi (Komfo Anokye Teaching Hospital)	233,62	Tamale Teaching Hospital	173,72
SAVANNAH	CENTRAL GONJA	Kumasi (Komfo Anokye Teaching Hospital)	249,97	Tamale Teaching Hospital	71,60
SAVANNAH	NORTH EAST GONJA	Kumasi (Komfo Anokye Teaching Hospital)	257,35	Tamale Teaching Hospital	82,45
SAVANNAH	WEST GONJA	Kumasi (Komfo Anokye Teaching Hospital)	281,22	Tamale Teaching Hospital	108,76
SAVANNAH	SAWLA-TUNA-KALBA	Kumasi (Komfo Anokye Teaching Hospital)	322,63	Tamale Teaching Hospital	167,41
SAVANNAH	NORTH GONJA	Kumasi (Komfo Anokye Teaching Hospital)	333,95	Tamale Teaching Hospital	83,78
UPPER EAST	BUILSA SOUTH	Kumasi (Komfo Anokye Teaching Hospital)	427,43	Tamale Teaching Hospital	136,92
UPPER EAST	BUILSA NORTH	Kumasi (Komfo Anokye Teaching Hospital)	446,86	Tamale Teaching Hospital	151,09
UPPER EAST	TALENSI	Kumasi (Komfo Anokye Teaching Hospital)	451,28	Tamale Teaching Hospital	140,13
UPPER EAST	KASENA NANKANA EAST	Kumasi (Komfo Anokye Teaching Hospital)	455,38	Tamale Teaching Hospital	151,90
UPPER EAST	BOLGATANGA MUNICIPAL	Kumasi (Komfo Anokye Teaching Hospital)	458,49	Tamale Teaching Hospital	151,57
UPPER EAST	BOLGA EAST	Kumasi (Komfo Anokye Teaching Hospital)	464,28	Tamale Teaching Hospital	154,66
UPPER EAST	KASENA NANKANA WEST	Kumasi (Komfo Anokye Teaching Hospital)	468,03	Tamale Teaching Hospital	172,24
UPPER EAST	NABDAM	Kumasi (Komfo Anokye Teaching Hospital)	471,66	Tamale Teaching Hospital	159,95
UPPER EAST	BAWKU WEST	Kumasi (Komfo Anokye Teaching Hospital)	476,09	Tamale Teaching Hospital	163,14
UPPER EAST	GARU	Kumasi (Komfo Anokye Teaching Hospital)	476,60	Tamale Teaching Hospital	164,52
UPPER EAST	BONGO	Kumasi (Komfo Anokye Teaching Hospital)	477,81	Tamale Teaching Hospital	168,37
UPPER EAST	BINDURI	Kumasi (Komfo Anokye Teaching Hospital)	494,04	Tamale Teaching Hospital	181,12
UPPER EAST	TEMPANE	Kumasi (Komfo Anokye Teaching Hospital)	498,14	Tamale Teaching Hospital	186,99

UPPER EAST	BAWKU MUNICIPAL	Kumasi (Komfo Anokye Teaching Hospital)	506,59	Tamale Teaching Hospital	193,85
**Region**	**District name**	**Radiotherapy facility before expansion**	**Euclidean Distance_1**	**Radiotherapy facility after expansion**	**Euclidean Distance_2**
UPPER EAST	PUSIGA	Kumasi (Komfo Anokye Teaching Hospital)	513,24	Tamale Teaching Hospital	201,18
UPPER WEST	WA WEST	Kumasi (Komfo Anokye Teaching Hospital)	371,94	Tamale Teaching Hospital	206,02
UPPER WEST	WA EAST	Kumasi (Komfo Anokye Teaching Hospital)	374,67	Tamale Teaching Hospital	149,68
UPPER WEST	WA MUNICIPAL	Kumasi (Komfo Anokye Teaching Hospital)	381,31	Tamale Teaching Hospital	191,59
UPPER WEST	NADOWLI-KALEO	Kumasi (Komfo Anokye Teaching Hospital)	413,51	Tamale Teaching Hospital	219,78
UPPER WEST	DAFFIAMA BUSSIE ISSA	Kumasi (Komfo Anokye Teaching Hospital)	416,79	Tamale Teaching Hospital	196,76
UPPER WEST	SISSALA EAST	Kumasi (Komfo Anokye Teaching Hospital)	439,80	Tamale Teaching Hospital	174,02
UPPER WEST	JIRAPA	Kumasi (Komfo Anokye Teaching Hospital)	444,23	Tamale Teaching Hospital	232,07
UPPER WEST	LAWRA	Kumasi (Komfo Anokye Teaching Hospital)	457,91	Tamale Teaching Hospital	257,87
UPPER WEST	SISSALA WEST	Kumasi (Komfo Anokye Teaching Hospital)	464,58	Tamale Teaching Hospital	223,21
UPPER WEST	LAMBUSSIE-KARNI	Kumasi (Komfo Anokye Teaching Hospital)	472,86	Tamale Teaching Hospital	251,55
UPPER WEST	NANDOM	Kumasi (Komfo Anokye Teaching Hospital)	478,57	Tamale Teaching Hospital	267,72
VOLTA	NORTH TONGU	Accra (Sweden Ghana Medical Centre)	88,25	Accra (Sweden Ghana Medical Centre)	88,25
VOLTA	SOUTH TONGU	Accra (Sweden Ghana Medical Centre)	105,30	Accra (Sweden Ghana Medical Centre)	105,30
VOLTA	CENTRAL TONGU	Accra (Sweden Ghana Medical Centre)	109,87	Accra (Sweden Ghana Medical Centre)	109,87
VOLTA	ANLOGA	Accra (Sweden Ghana Medical Centre)	115,15	Accra (Sweden Ghana Medical Centre)	115,15
VOLTA	SOUTH DAYI	Accra (Sweden Ghana Medical Centre)	124,13	Accra (Sweden Ghana Medical Centre)	124,13
VOLTA	AKATSI SOUTH	Accra (Sweden Ghana Medical Centre)	126,74	Accra (Sweden Ghana Medical Centre)	126,74
VOLTA	ADAKLU	Accra (Sweden Ghana Medical Centre)	126,93	Accra (Sweden Ghana Medical Centre)	126,93
VOLTA	HO WEST	Accra (Sweden Ghana Medical Centre)	127,38	Accra (Sweden Ghana Medical Centre)	127,38
VOLTA	KETA MUNICIPAL	Accra (Sweden Ghana Medical Centre)	129,77	Accra (Sweden Ghana Medical Centre)	129,77
VOLTA	AKATSI NORTH	Accra (Sweden Ghana Medical Centre)	142,03	Accra (Sweden Ghana Medical Centre)	142,03

VOLTA	AGOTIME ZIOPE	Accra (Sweden Ghana Medical Centre)	142,54	Accra (Sweden Ghana Medical Centre)	142,54
**Region**	**District name**	**Radiotherapy facility before expansion**	**Euclidean Distance_1**	**Radiotherapy facility after expansion**	**Euclidean Distance_2**
VOLTA	KETU NORTH	Accra (Sweden Ghana Medical Centre)	146,02	Accra (Sweden Ghana Medical Centre)	146,02
VOLTA	HO MUNICIPAL	Accra (Sweden Ghana Medical Centre)	146,58	Accra (Sweden Ghana Medical Centre)	146,58
VOLTA	NORTH DAYI	Accra (Sweden Ghana Medical Centre)	148,65	Accra (Sweden Ghana Medical Centre)	148,65
VOLTA	KETU SOUTH	Accra (Sweden Ghana Medical Centre)	154,12	Accra (Sweden Ghana Medical Centre)	154,12
VOLTA	AFADZATO SOUTH	Accra (Sweden Ghana Medical Centre)	164,26	Accra (Sweden Ghana Medical Centre)	164,26
VOLTA	KPANDO MUNICIPAL	Accra (Sweden Ghana Medical Centre)	170,08	Accra (Sweden Ghana Medical Centre)	170,08
VOLTA	HOHOE MUNICIPAL	Accra (Sweden Ghana Medical Centre)	193,73	Accra (Sweden Ghana Medical Centre)	193,73
WESTERN	WASSA AMENFI EAST	Kumasi (Komfo Anokye Teaching Hospital)	104,60	Kumasi (Komfo Anokye Teaching Hospital)	104,60
WESTERN	WASSA AMENFI CENTRAL	Kumasi (Komfo Anokye Teaching Hospital)	128,57	Kumasi (Komfo Anokye Teaching Hospital)	128,57
WESTERN	WASSA AMENFI WEST	Kumasi (Komfo Anokye Teaching Hospital)	143,18	Kumasi (Komfo Anokye Teaching Hospital)	143,18
WESTERN	PRESTEA/HUNI VALLEY	Kumasi (Komfo Anokye Teaching Hospital)	143,82	Kumasi (Komfo Anokye Teaching Hospital)	143,82
WESTERN	WASSA EAST	Kumasi (Komfo Anokye Teaching Hospital)	149,71	Kumasi (Komfo Anokye Teaching Hospital)	149,71
WESTERN	SHAMA	Accra (Korle Bu Teaching Hospital)	167,62	Accra (Korle Bu Teaching Hospital)	167,62
WESTERN	TARKWA NSUAEM	Kumasi (Komfo Anokye Teaching Hospital)	176,73	Kumasi (Komfo Anokye Teaching Hospital)	176,73
WESTERN	SEKONDI TAKORADI METROPOLIS	Accra (Korle Bu Teaching Hospital)	180,34	Accra (Korle Bu Teaching Hospital)	180,34
WESTERN	MPOHOR	Kumasi (Komfo Anokye Teaching Hospital)	183,21	Kumasi (Komfo Anokye Teaching Hospital)	183,21
WESTERN	EFFIA KWESIMINTSIM MUNICIPAL	Accra (Korle Bu Teaching Hospital)	186,20	Accra (Korle Bu Teaching Hospital)	186,20
WESTERN	NZEMA EAST	Kumasi (Komfo Anokye Teaching Hospital)	188,11	Kumasi (Komfo Anokye Teaching Hospital)	188,11
WESTERN	ELLEMBELLE	Kumasi (Komfo Anokye Teaching Hospital)	194,12	Kumasi (Komfo Anokye Teaching Hospital)	194,12
WESTERN	JOMORO	Kumasi (Komfo Anokye Teaching Hospital)	204,77	Kumasi (Komfo Anokye Teaching Hospital)	204,77
WESTERN	AHANTA WEST MUNICIPAL	Kumasi (Komfo Anokye Teaching Hospital)	208,31	Kumasi (Komfo Anokye Teaching Hospital)	208,31
WESTERN NORTH	BIBIANI-ANHWIASO-	Kumasi (Komfo Anokye Teaching Hospital)	83,49	Kumasi (Komfo Anokye Teaching Hospital)	83,49

© 2022 Scott AA et al. *JAMA Network Open.*

	BEKWAI MUNICIPAL				
WESTERN NORTH	SEFWI-WIAWSO	Kumasi (Komfo Anokye Teaching Hospital)	108,63	Kumasi (Komfo Anokye Teaching Hospital)	108,63
**Region**	**District name**	**Radiotherapy facility before expansion**	**Euclidean Distance_1**	**Radiotherapy facility after expansion**	**Euclidean Distance_2**
WESTERN NORTH	SEFWI AKONTOMBRA	Kumasi (Komfo Anokye Teaching Hospital)	135,95	Kumasi (Komfo Anokye Teaching Hospital)	135,95
WESTERN NORTH	BODI	Kumasi (Komfo Anokye Teaching Hospital)	140,80	Kumasi (Komfo Anokye Teaching Hospital)	140,80
WESTERN NORTH	JUABOSO	Kumasi (Komfo Anokye Teaching Hospital)	146,25	Kumasi (Komfo Anokye Teaching Hospital)	146,25
WESTERN NORTH	BIA EAST	Kumasi (Komfo Anokye Teaching Hospital)	153,21	Kumasi (Komfo Anokye Teaching Hospital)	153,21
WESTERN NORTH	BIA WEST	Kumasi (Komfo Anokye Teaching Hospital)	162,43	Kumasi (Komfo Anokye Teaching Hospital)	162,43
WESTERN NORTH	AOWIN	Kumasi (Komfo Anokye Teaching Hospital)	162,96	Kumasi (Komfo Anokye Teaching Hospital)	162,96
WESTERN NORTH	SUAMAN	Kumasi (Komfo Anokye Teaching Hospital)	167,39	Kumasi (Komfo Anokye Teaching Hospital)	167,39

© 2022 Scott AA et al. *JAMA Network Open.*

eFigure. Distance From District Centroid to Nearest Radiotherapy Facility

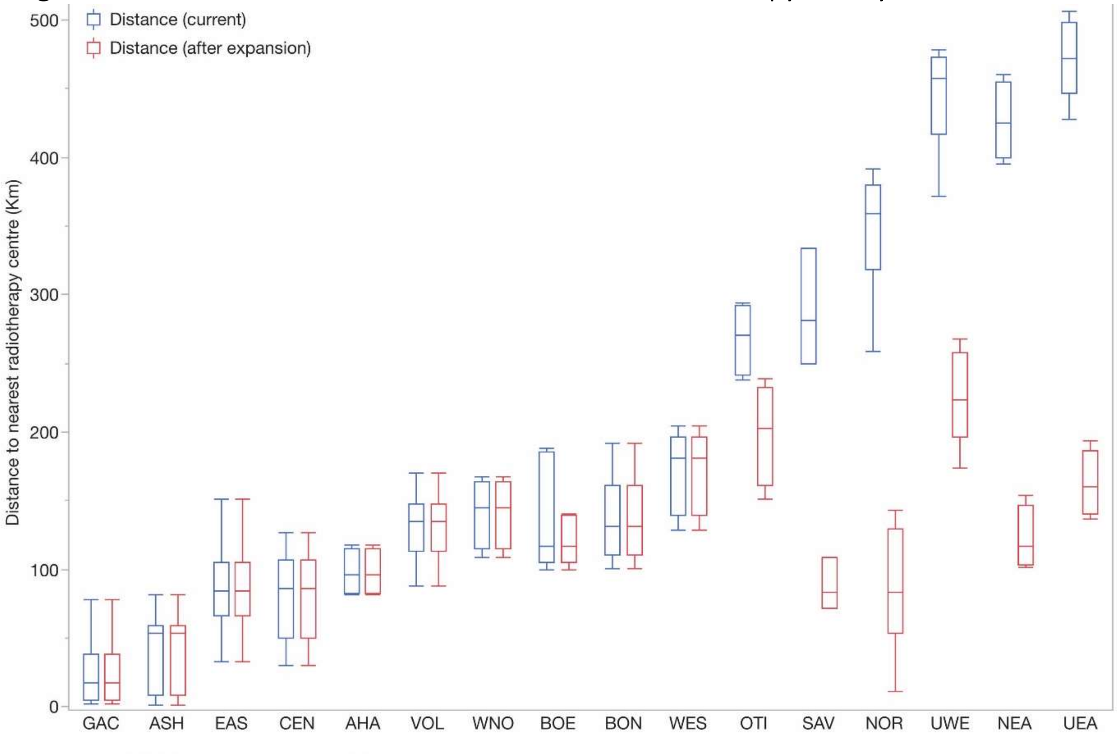

This visualization is built with a random sample of 130 rows.

© 2022 Scott AA et al. *JAMA Network Open.*
